# Quantitative assessment of myocardial motion from velocity encoded MRI

**DOI:** 10.1186/1532-429X-14-S1-W39

**Published:** 2012-02-01

**Authors:** Anja Lutz, Jan Paul, Patrick Etyngier, Axel Bornstedt, Peter Bernhardt, Gerd Ulrich Nienhaus, Wolfgang Rottbauer, Volker Rasche

**Affiliations:** 1 Department of Internal Medicine II, University Hospital of Ulm, Ulm, Germany; 2 Institute of Applied Physics and Center for Functional Nanostructures, Karlsruhe Institute of Technology, Karlsruhe, Germany; 3 Medisys Research Lab, Philips Healthcare, Suresnes, France

## Summary

It is objective of this study to investigate the potential role of different automatically derived quantitative parameters derived from velocity encoded MRI for the identification of asynchronic patients.

## Background

About 30% of patients treated with cardiac resynchronization therapy (CRT) do not benefit from the procedure. Quantitative analysis of motion encoded MRI data may provide helpful parameters for the identification of CRT patients and prediction of the therapy outcome.

## Methods

11 Volunteers (30±8 years) and 3 patients (41±11 years) were investigated at a 3T whole body MR scanner (Achieva, Philips) with a 32 channel cardiac coil. The patients suffered from DCM, asynchrony and/or LBBB. A velocity encoded (TPM) navigated segmented gradient echo sequence was applied in the apical, equatorial and basal slice. The acquisition parameters were: FOV=340^2^mm^2^, in-plane resolution =2.5^2^mm^2^, slice thickness=8mm, acquisition matrix MxP=172x168, TR/TE=6.3ms/4.6ms, α=15°, 3 k-lines per segment, VENC=30cm/s, nominal scan duration =5:51 minutes, black blood imaging with alternating presaturation pulses [1] and a SENSE acceleration factor of 2. For 60 bpm 32 cardiac phases were measured with a phase interval of 29.1ms.

From the TPM data, the longitudinal and radial standard deviation of time to peak systolic and diastolic velocities SD(TTP_l,sys_), SD(TTP_l,dias_), SD(TTP_r,sys_), SD(TTP_r,dias_)over 6 segments [2], the radial, circumferential and longitudinal asynchrony correlation coefficient (ACC)[3], the longitudinal and radial velocity range Δv_l_ = v_l,max_-v_l,min_, Δv_r_ = v_r,max_-v_r,min_ and the temporal uniformity of velocity (TUV) in radial, longitudinal and circumferential direction were derived. The latter one was defined in analogy to the temporal uniformity of strain[4,5].

## Results

**Figure 1:**
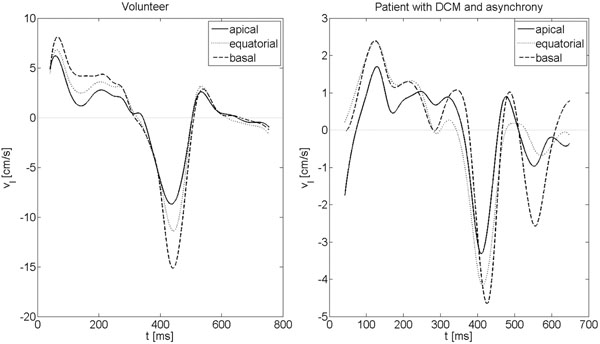
Apical, equatorial and basal longitudinal motion exemplarily for one volunteer.

In all patients, a substantially detoriation of the motion curve from the healthy volunteers was observed (Figure 1). Most obvious, a clear reduction of the peak velocities can be appreciated.

The derived quantitative motion parameters are listed in table 1. All mean values of the derived parameters show differences between the patient and the volunteer group. Δv_l_ and Δv_r_ show large differences for all investigated motion directions, SD(TTP) appears increased, the mean ACC and the radial and longitudinal TUV reduced. Significances of the differences could not be calculated due to the small number of patients.

**Table 1:** Velocity based motion parameters evaluated for all volunteers (mean ± standard deviation) as well for the 3 investigated patients.

	volunteers		patients	
parameter	mean	σ	mean	σ

SD(TTP_l,sys_ [ms]	36.84	22.53	48.94	27.77
SD(TTP_l,dias_ [ms]	15.72	4.64	45.68	22.23
SD(TTP_r,sys_ [ms]	46.40	12.48	61.78	17.07
SD(TTP_l,dias_ [ms]	33.85	8.75	49.75	21.39
mean ACC_l_	0.93	0.03	0.70	0.09
mean ACC_r_	0.79	0.04	0.52	0.07
mean ACC_c_	0.72	0.07	0.53	0.16
Δv_l,apical_ [cm/s]	10.77	2.19	5.12	1.37
Δv_l,equatorial_ [cm/s]	16.13	2.54	7.14	3.27
Δv_l,basal_ [cm/s]	20.71	2.50	7.26	3.11
Δv_r,apical_ [cm/s]	8.31	1.05	4.51	1.21
Δv_r,equatorial_ [cm/s]	7.97	0.97	4.26	0.40
Δv_r,basal_ [cm/s]	7.51	1.23	5.15	1.04
TUV_l_	0.87	0.02	0.78	0.04
TUV_r_	0.80	0.02	0.70	0.02
TUV_c_	0.77	0.03	0.69	0.06

## Conclusions

Several quantitative motion parameters show substantial differences between patients and volunteers and may be applied for automatic identification left ventricular asynchrony. Whether the investigated parameters can be applied for CRT patient selection and outcome prediction must be proven in a larger clinical study.

## Funding

AL and VR have a research agreement with Philips Medical. PE is employed by Philips Healthcare.
